# The effects of multiple features of alternatively spliced exons on the *K*_*A*_*/K*_*S *_ratio test

**DOI:** 10.1186/1471-2105-7-259

**Published:** 2006-05-19

**Authors:** Feng-Chi Chen, Trees-Juen Chuang

**Affiliations:** 1 Genomics Research Center, Academia Sinica, Academia Road, Nankang, Taipei 11529, Taiwan; 2 Division of Biostatistics and Bioinformatics, National Health Research Institute, Miaoli County 350, Taiwan

## Abstract

**Background:**

The evolution of alternatively spliced exons (ASEs) is of primary interest because these exons are suggested to be a major source of functional diversity of proteins. Many exon features have been suggested to affect the evolution of ASEs. However, previous studies have relied on the *K*_*A*_*/K*_*S *_ratio test without taking into consideration information sufficiency (i.e., exon length > 75 bp, cross-species divergence > 5%) of the studied exons, leading to potentially biased interpretations. Furthermore, which exon feature dominates the results of the *K*_*A*_*/K*_*S *_ratio test and whether multiple exon features have additive effects have remained unexplored.

**Results:**

In this study, we collect two different datasets for analysis – the ASE dataset (which includes lineage-specific ASEs and conserved ASEs) and the ACE dataset (which includes only conserved ASEs). We first show that information sufficiency can significantly affect the interpretation of relationship between exons features and the *K*_*A*_*/K*_*S *_ratio test results. After discarding exons with insufficient information, we use a Boolean method to analyze the relationship between test results and four exon features (namely length, protein domain overlapping, inclusion level, and exonic splicing enhancer (ESE) frequency) for the ASE dataset. We demonstrate that length and protein domain overlapping are dominant factors, and they have similar impacts on test results of ASEs. In addition, despite the weak impacts of inclusion level and ESE motif frequency when considered individually, combination of these two factors still have minor additive effects on test results. However, the ACE dataset shows a slightly different result in that inclusion level has a marginally significant effect on test results. Lineage-specific ASEs may have contributed to the difference. Overall, in both ASEs and ACEs, protein domain overlapping is the most dominant exon feature while ESE frequency is the weakest one in affecting test results.

**Conclusion:**

The proposed method can easily find additive effects of individual or multiple factors on the *K*_*A*_*/K*_*S *_ratio test results of exons. Therefore, the system can analyze complex conditions in evolution where multiple features are involved. More factors can also be added into the system to extend the scope of evolutionary analysis of exons. In addition, our method may be useful when orthologous exons can not be found for the *K*_*A*_*/K*_*S *_ratio test.

## Background

Alternative splicing (AS) is suggested to be a mechanism to relax selection pressure [1-3]. It allows generation of different transcript/protein isoforms from the same genes, leading to increased functional diversity of the proteome. The evolution of alternatively spliced exons (ASEs) has been a topic of extensive studies. A number of exon features have been reported to influence the evolutionary rates of ASEs, such as length [4] and inclusion level (defined as the fraction of ESTs that include a certain exon) [2,5-11]. Previous studies used the non-synonymous to synonymous substitution rate (*K*_*A*_*/K*_*S*_) ratio test to evaluate the relationships between exon features and evolutionary rates of ASEs. The *K*_*A*_*/K*_*S *_ratio test is frequently utilized to examine the evolutionary rates of ASEs. Coding exons are regarded under strong negative selection if they pass the test (i.e. *K*_*A*_*/K*_*S *_ratio significantly smaller than one [12,13]). Therefore, the proportion of ASEs that fail the test (i.e. failing-test exon proportion, or FTE proportion) can be used to indicate the strength of selection pressure and the level of amino acid changes normalized by synonymous substation rate. It was suggested that exons ≤ 75 bp or has ≤ 5% nucleotide substitution rate between species (collectively we call these two exon types "non-applicable exons") may contain insufficient information, rendering the *K*_*A*_*/K*_*S *_ratio test powerless [12]. However, previous studies did not take into account the limitations of the *K*_*A*_*/K*_*S *_ratio test. Since ASEs tend to be short in length and have small genetic distances [14-18], a large portion of ASEs is "non-applicable". Inclusion of non-applicable ASEs may result in high FTE proportion [12] and lead to questionable inferences of ASE evolution. Therefore, it is necessary to re-examine the relationships between inclusion level/length and evolutionary rates of ASEs using only "applicable" exons.

Furthermore, the evolutionary rates of ASEs may be simultaneously affected by multiple factors. The relative strength of individual factors and additive effects of multiple factors on ASE evolution have not been systematically explored. Two factors other than length and inclusion level may also affect the evolution of ASEs: protein domain overlapping and frequency of exonic splicing enhancers (ESEs). Domain overlapping is important because functional domains are suggested to be under strong selection pressure [1,17,19,20]. Meanwhile, ESEs are *cis*-regulatory elements that regulate pre-mRNA splicing [18,21,22]. The conservation of ESE motifs in ASEs supposedly would reduce the evolutionary rates in these exons.

In this study, we would like to address the following questions: (1) Which of the four factors stated above has the greatest effect on the evolutionary rates of ASEs? (2) Are there additive effects between these factors? (3) What are the combinations of these factors that make ASEs most conserved? We first examine whether non-applicable exons affect the interpretations of the exon feature-FTE proportion relationships. Then we design a Boolean function combined with the Karnaugh map [23] to represent the evolutionary effects of combinational factors (or multiple factors) and to determine which conditions have dominant (or powerless) impacts on the FTE proportion of ASEs. Furthermore, since splicing patterns may differ between human and mouse, the mouse orthologues of human ASEs can be in fact constitutively spliced exons (CSEs). Therefore, we collected two datasets – the ASE dataset and the ACE (conserved ASEs or alternative conserved exons defined in [17]) dataset – for testing the effects of exon features on evolution (see Methods for more details).

## Results and discussion

### Non-applicable exons significantly affect the relationship between FTE proportion and inclusion level/length of ASEs

The basic features of studied human CSEs (4630 exons) and ASEs (508 major form exons and 270 non-major-form exons, see Methods for definition) are listed in Table 1. It appears that ASEs include more short exons and more low-divergence exons than CSEs (*P*-value < 0.01 by Fisher's exact test). Therefore, it is clear that ASEs include a much higher proportion of non-applicable exons than CSEs (*P*-value < 10^-6^).

**Figure 1:**
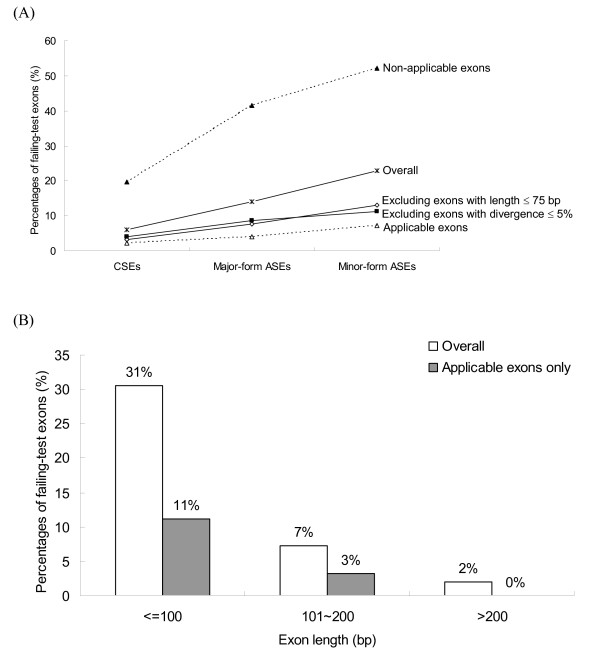
The relationship between FTE proportion and (A) inclusion level; and (B) length of ASEs. It is clear that non-applicable exons have significantly higher FTE proportions than the rest of the exons. Therefore, non-applicable exons can bias interpretations of the *K*_*A*_*/K*_*S *_ratio test results.

The FTE proportions of CSEs, major-form ASEs, and non-major-form ASEs are shown in Figure 1a. When all exons are considered, inclusion level has a clear negative relationship with FTE proportion. Both the FTE proportions of major-form ASEs and non-major-form ASEs are significantly higher than that of CSEs (*P*-value < 10^-9 ^and *P*-value < 10^-17^, respectively). We then try to exclude non-applicable exons and re-analyze the new dataset. It appears that exclusion of these exons decreases the FTE proportions in all three exon types while the negative relationship between inclusion level and FTE proportion remains (Fig. 1A). However, the relationship is remarkably weakened (*P*-values > 0.05 for both CSE vs. major-form and major-form vs. non-major-form). Also worth noting is that the FTE proportions in all three exon types have fallen short of 9% (the genome-wide average of FTE proportion [12]), indicating that even for non-major-from exons, the *K*_*A*_*/K*_*S *_ratio test is an adequate prediction tool as long as non-applicable exons are excluded. In addition, Figure 1A also shows that non-applicable exons have a very high proportion of FTEs, even for CSEs (20% of non-applicable CSEs fail the test). Surprisingly, more than 40% of non-applicable ASEs fail the *K*_*A*_*/K*_*S *_ratio test. The differences in FTE proportion between overall and applicable exons are highly significant regardless of inclusion level (*P*-value < 10^-4 ^for all three exon types). Overall, the inclusion level of ASEs appears to be weakly associated with the strength of selection pressure. The weakened relationship between inclusion level and percentage of FTEs when non-applicable exons are excluded implies that inclusion level may not be the most important factor that affects evolutionary rates of ASEs.

On the other hand, FTE proportion reduces remarkably when the lengths of ASEs exceed 100 bp (Figure 1B). The difference in FTE proportion between short ASEs (≤ 100 bp) and longer ASEs (> 100 bp) is highly significant (*P*-value < 0.001) even when non-applicable exons are excluded. Furthermore, the FTE proportion is as high as 75% for ASEs with length ≤ 50 bp (data not shown). In comparison, only 11% of short, applicable ASEs fail the test (Figure 1B). The difference in FTE proportion between all short exons (31%) and short, applicable exons (11%) is highly significant (*P*-value < 10^-5^). Therefore, although the negative relationship between length and FTE proportion remains significant after excluding non-applicable exons, the applicability of exons still has remarkable influences on the FTE proportions of ASEs, particularly for short ASEs. Overall, our results reveal that interpretations of the relationships between FTE proportion and exon features without excluding non-applicable exons may be misleading. As a result, the following analysis includes only applicable exons.

### Influences of protein domain and ESE motif frequency on the FTE proportions of ASEs

**Figure 2:**
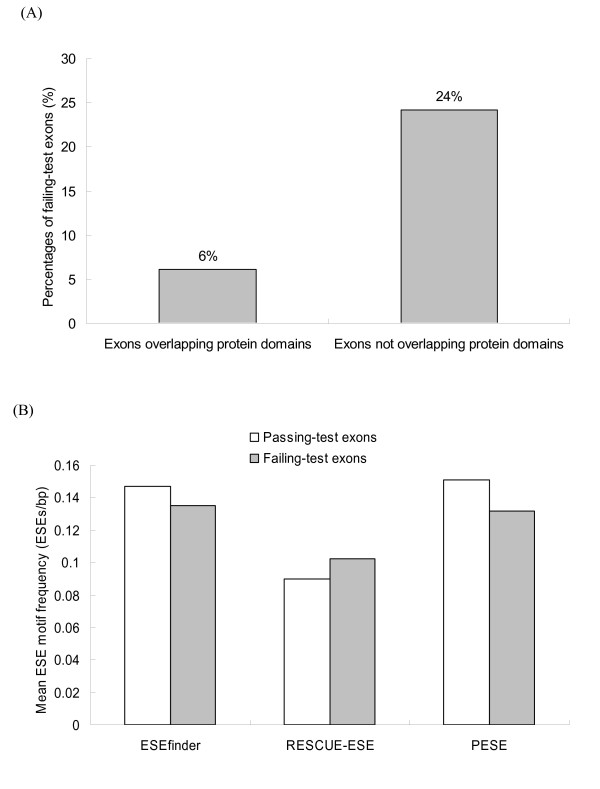
(A) The relationship between FTE proportion and protein domain overlapping; (B) Mean ESE motif frequencies in passing-test and failing-test ASEs. Note that only applicable ASEs are considered here.

Since functional protein domains are usually under selection pressure [1,17,19,20], ASEs overlapping protein domains may be well conserved. The InterProScan package and the INTERPRO resource [24,25] (downloaded from [26,27]) are used for protein domain prediction. An ASE is designated as "overlapping with protein domain" if at least 30 amino acids of the ASE can be found in an Interpro-predicted domain. Figure 2A shows that ASEs that overlap with protein domains have a much lower FTE proportion than those that do not (6% vs. 24%, *P*-values < 10^-4 ^by the two-tailed Fisher's exact test). Therefore, our results suggest that protein domain overlapping has a significant effect on the evolution of ASEs.

Meanwhile, we used three packages: ESEfinder [28], RESCUE-ESE [29-31], and PESE [32,33], to identify ESEs in the studied ASEs. Figure 2B illustrates that the mean ESE motif frequencies identified by the three packages in passing-test and failing-test ASEs are somewhat different. However, none of the three differences is significant (all *P *> 0.1 by the Mann-Whitney U-test). Consequently, the observation implies that ESE motif frequencies are only weakly related to FTE proportions of ASEs. This unexpected result may have resulted from overestimation of ESE motifs by the ESE prediction programs [34]. Alternatively, it is also possible that ESEs in fact are not under very strong selection pressure because intronic regulatory elements may also participate in splicing regulation [16,17,34-36].

### Influences of single and multiple factors on evolutionary rates of ASEs

So far we have discussed the influences of single factors on the evolution of ASEs. It is of interest to explore the following questions: (i) which one of the four factors mentioned above has the largest influence on FTE proportion; and (ii) how do combinations of these factors affect FTE proportion. For simplicity, we denote these four factors as *A *(length)*, B *(protein domain overlapping)*, C *(inclusion level), and *D *(ESE motif frequency), and define four Boolean functions *f*_*A*_(*e*_*i*_), *f*_*B*_(*e*_*i*_), *f*_*C*_(*e*_*i*_), and *f*_*D*_(*e*_*i*_) for each exon *e*_*i *_as follows:

fA(ei)={0,if length of ei≤100 bp (short exon)1,if length of ei>100 bp     (1)
 MathType@MTEF@5@5@+=feaafiart1ev1aaatCvAUfKttLearuWrP9MDH5MBPbIqV92AaeXatLxBI9gBaebbnrfifHhDYfgasaacH8akY=wiFfYdH8Gipec8Eeeu0xXdbba9frFj0=OqFfea0dXdd9vqai=hGuQ8kuc9pgc9s8qqaq=dirpe0xb9q8qiLsFr0=vr0=vr0dc8meaabaqaciaacaGaaeqabaqabeGadaaakeaacqWGMbGzdaWgaaWcbaGaemyqaeeabeaakiabcIcaOiabdwgaLnaaBaaaleaacqWGPbqAaeqaaOGaeiykaKIaeyypa0ZaaiqaaeaafaqaaeGacaaabaGaeGimaaJaeiilaWcabaGaeeyAaKMaeeOzayMaeeiiaaIaeeiBaWMaeeyzauMaeeOBa4Maee4zaCMaeeiDaqNaeeiAaGMaeeiiaaIaee4Ba8MaeeOzayMaeeiiaaIaeeyzau2aaSbaaSqaaGqaaiab=LgaPbqabaGccqGHKjYOcqaIXaqmcqaIWaamcqaIWaamcqqGGaaicqqGIbGycqqGWbaCcqqGGaaicqGGOaakcqqGZbWCcqqGObaAcqqGVbWBcqqGYbGCcqqG0baDcqqGGaaicqqGLbqzcqqG4baEcqqGVbWBcqqGUbGBcqGGPaqkaeaacqaIXaqmcqGGSaalaeaacqqGPbqAcqqGMbGzcqqGGaaicqqGSbaBcqqGLbqzcqqGUbGBcqqGNbWzcqqG0baDcqqGObaAcqqGGaaicqqGVbWBcqqGMbGzcqqGGaaicqqGLbqzdaWgaaWcbaGae8xAaKgabeaakiabg6da+iabigdaXiabicdaWiabicdaWiabbccaGiabbkgaIjabbchaWbaaaiaawUhaaiaaxMaacaWLjaWaaeWaaeaacqaIXaqmaiaawIcacaGLPaaaaaa@820F@

fB(ei)={0,if ei does not overlap with protein domains1,if ei overlaps with protein domains     (2)
 MathType@MTEF@5@5@+=feaafiart1ev1aaatCvAUfKttLearuWrP9MDH5MBPbIqV92AaeXatLxBI9gBaebbnrfifHhDYfgasaacH8akY=wiFfYdH8Gipec8Eeeu0xXdbba9frFj0=OqFfea0dXdd9vqai=hGuQ8kuc9pgc9s8qqaq=dirpe0xb9q8qiLsFr0=vr0=vr0dc8meaabaqaciaacaGaaeqabaqabeGadaaakeaacqWGMbGzdaWgaaWcbaGaemOqaieabeaakiabcIcaOiabdwgaLnaaBaaaleaacqWGPbqAaeqaaOGaeiykaKIaeyypa0ZaaiqaaeaafaqaaeGacaaabaGaeGimaaJaeiilaWcabaGaeeyAaKMaeeOzayMaeeiiaaIaeeyzau2aaSbaaSqaaGqaaiab=LgaPbqabaGccqqGGaaicqqGKbazcqqGVbWBcqqGLbqzcqqGZbWCcqqGGaaicqqGUbGBcqqGVbWBcqqG0baDcqqGGaaicqqGVbWBcqqG2bGDcqqGLbqzcqqGYbGCcqqGSbaBcqqGHbqycqqGWbaCcqqGGaaicqqG3bWDcqqGPbqAcqqG0baDcqqGObaAcqqGGaaicqqGWbaCcqqGYbGCcqqGVbWBcqqG0baDcqqGLbqzcqqGPbqAcqqGUbGBcqqGGaaicqqGKbazcqqGVbWBcqqGTbqBcqqGHbqycqqGPbqAcqqGUbGBcqqGZbWCaeaacqaIXaqmcqGGSaalaeaacqqGPbqAcqqGMbGzcqqGGaaicqqGLbqzdaWgaaWcbaGae8xAaKgabeaakiabbccaGiabb+gaVjabbAha2jabbwgaLjabbkhaYjabbYgaSjabbggaHjabbchaWjabbohaZjabbccaGiabbEha3jabbMgaPjabbsha0jabbIgaOjabbccaGiabbchaWjabbkhaYjabb+gaVjabbsha0jabbwgaLjabbMgaPjabb6gaUjabbccaGiabbsgaKjabb+gaVjabb2gaTjabbggaHjabbMgaPjabb6gaUjabbohaZbaacaWLjaGaaCzcamaabmaabaGaeGOmaidacaGLOaGaayzkaaaacaGL7baaaaa@A1CF@

fC(ei)={0,if ei∈non-major form exons1,if ei∈major-form exons     (3)
 MathType@MTEF@5@5@+=feaafiart1ev1aaatCvAUfKttLearuWrP9MDH5MBPbIqV92AaeXatLxBI9gBaebbnrfifHhDYfgasaacH8akY=wiFfYdH8Gipec8Eeeu0xXdbba9frFj0=OqFfea0dXdd9vqai=hGuQ8kuc9pgc9s8qqaq=dirpe0xb9q8qiLsFr0=vr0=vr0dc8meaabaqaciaacaGaaeqabaqabeGadaaakeaacqWGMbGzdaWgaaWcbaGaem4qameabeaakiabcIcaOiabdwgaLnaaBaaaleaacqWGPbqAaeqaaOGaeiykaKIaeyypa0ZaaiqaaeaafaqaaeGacaaabaGaeGimaaJaeiilaWcabaGaeeyAaKMaeeOzayMaeeiiaaIaeeyzau2aaSbaaSqaaGqaaiab=LgaPbqabaGccqGHiiIZcqqGUbGBcqqGVbWBcqqGUbGBcqqGTaqlcqqGTbqBcqqGHbqycqqGQbGAcqqGVbWBcqqGYbGCcqqGGaaicqqGMbGzcqqGVbWBcqqGYbGCcqqGTbqBcqqGGaaicqqGLbqzcqqG4baEcqqGVbWBcqqGUbGBcqqGZbWCaeaacqaIXaqmcqGGSaalaeaacqqGPbqAcqqGMbGzcqqGGaaicqqGLbqzdaWgaaWcbaGae8xAaKgabeaakiabgIGiolabb2gaTjabbggaHjabbQgaQjabb+gaVjabbkhaYjabb2caTiabbAgaMjabb+gaVjabbkhaYjabb2gaTjabbccaGiabbwgaLjabbIha4jabb+gaVjabb6gaUjabbohaZbaaaiaawUhaaiaaxMaacaWLjaWaaeWaaeaacqaIZaWmaiaawIcacaGLPaaaaaa@7C0B@

fD(ei)={0,if ei with low ESE motif frequency1,if ei with high ESE motif frequency     (4)
 MathType@MTEF@5@5@+=feaafiart1ev1aaatCvAUfKttLearuWrP9MDH5MBPbIqV92AaeXatLxBI9gBaebbnrfifHhDYfgasaacH8akY=wiFfYdH8Gipec8Eeeu0xXdbba9frFj0=OqFfea0dXdd9vqai=hGuQ8kuc9pgc9s8qqaq=dirpe0xb9q8qiLsFr0=vr0=vr0dc8meaabaqaciaacaGaaeqabaqabeGadaaakeaacqWGMbGzdaWgaaWcbaGaemiraqeabeaakiabcIcaOiabdwgaLnaaBaaaleaacqWGPbqAaeqaaOGaeiykaKIaeyypa0ZaaiqaaeaafaqaaeGacaaabaGaeGimaaJaeiilaWcabaGaeeyAaKMaeeOzayMaeeiiaaIaeeyzau2aaSbaaSqaaGqaaiab=LgaPbqabaGccqqGGaaiieGacqGF3bWDcqqGPbqAcqqG0baDcqqGObaAcqqGGaaicqqGSbaBcqqGVbWBcqqG3bWDcqqGGaaicqqGfbqrcqqGtbWucqqGfbqrcqqGGaaicqqGTbqBcqqGVbWBcqqG0baDcqqGPbqAcqqGMbGzcqqGGaaicqqGMbGzcqqGYbGCcqqGLbqzcqqGXbqCcqqG1bqDcqqGLbqzcqqGUbGBcqqGJbWycqqG5bqEaeaacqaIXaqmcqGGSaalaeaacqqGPbqAcqqGMbGzcqqGGaaicqqGLbqzdaWgaaWcbaGae8xAaKgabeaakiabbccaGiab+Dha3jabbMgaPjabbsha0jabbIgaOjabbccaGiabbIgaOjabbMgaPjabbEgaNjabbIgaOjabbccaGiabbweafjabbofatjabbweafjabbccaGiabb2gaTjabb+gaVjabbsha0jabbMgaPjabbAgaMjabbccaGiabbAgaMjabbkhaYjabbwgaLjabbghaXjabbwha1jabbwgaLjabb6gaUjabbogaJjabbMha5baaaiaawUhaaiaaxMaacaWLjaWaaeWaaeaacqaI0aanaiaawIcacaGLPaaaaaa@93C7@

where the definitions of low/high ESE motif frequency is given in Methods.

Table 2 illustrates the impact of each single factor on the FTE proportions of ASEs. It reveals that short exons have the highest proportion of FTEs (11.2%), followed by exons that do not overlap protein domains (8.8%), non-major-form exons (7.3%), and lastly by exons with low ESE motif frequency (6.4%). Factors A and B are significantly related to FTE proportion (*P *< 0.001, all tests used in the section are the two-tailed Fisher's exact test), whereas factors C and D are not (*P *> 0.1). To determine which of A and B impacts the *K*_*A*_*/K*_*S *_ratio more, we compared the FTE proportions of two different combinations of the two factors. Table 2 shows that long exons without overlapping protein domains have a slightly higher FTE proportion than short exons that overlap protein domains (5.8% vs. 4.0%). However, the difference is not significant (*P *> 0.1). Therefore, we suggest that length and protein domain overlapping have similar impacts on FTE proportions of ASEs.

We then consider the additive impacts of the four factors on FTE proportion. According to Equations (1) ~ (4), there are 2^4 ^(= 16) possible combinations (or patterns) for each exon e_*i*_, assigned as (*f*_*A*_(*e*_*i*_), *f*_*B*_(*e*_*i*_), *f*_*C*_(*e*_*i*_), *f*_*D*_(*e*_*i*_)). For simplicity, these 16 groups are denoted as

*g*_*j*_(*A*,*B*,*C*,*D*), ∀ *A*, *B*, *C*, *D *∈ {0,1} and *j *= ((((*A*×2)+*B*)×2)+*C*)×2+*D *    (5)

These 16 groups therefore include *g*_0_(0,0,0,0), *g*_1_(0,0,0,1),......, *g*_15_(1,1,1,1). Using Boolean algebraic expression, they are also denoted as *g*_0 _(a¯,b¯,c¯,d¯
 MathType@MTEF@5@5@+=feaafiart1ev1aaatCvAUfKttLearuWrP9MDH5MBPbIqV92AaeXatLxBI9gBaebbnrfifHhDYfgasaacH8akY=wiFfYdH8Gipec8Eeeu0xXdbba9frFj0=OqFfea0dXdd9vqai=hGuQ8kuc9pgc9s8qqaq=dirpe0xb9q8qiLsFr0=vr0=vr0dc8meaabaqaciaacaGaaeqabaqabeGadaaakeaacuWGHbqygaqeaiabcYcaSiqbdkgaIzaaraGaeiilaWIafm4yamMbaebacqGGSaalcuWGKbazgaqeaaaa@34E4@) *g*_1 _(a¯,b¯,c¯
 MathType@MTEF@5@5@+=feaafiart1ev1aaatCvAUfKttLearuWrP9MDH5MBPbIqV92AaeXatLxBI9gBaebbnrfifHhDYfgasaacH8akY=wiFfYdH8Gipec8Eeeu0xXdbba9frFj0=OqFfea0dXdd9vqai=hGuQ8kuc9pgc9s8qqaq=dirpe0xb9q8qiLsFr0=vr0=vr0dc8meaabaqaciaacaGaaeqabaqabeGadaaakeaacuWGHbqygaqeaiabcYcaSiqbdkgaIzaaraGaeiilaWIafm4yamMbaebaaaa@329B@,*d*),......, *g*_15_(*a*,*b*,*c*,*d*), where *a *(or *b *or *c *or *d*) stands for *A *(*B *or *C *or *D*) = 1 and a¯
 MathType@MTEF@5@5@+=feaafiart1ev1aaatCvAUfKttLearuWrP9MDH5MBPbIqV92AaeXatLxBI9gBaebbnrfifHhDYfgasaacH8akY=wiFfYdH8Gipec8Eeeu0xXdbba9frFj0=OqFfea0dXdd9vqai=hGuQ8kuc9pgc9s8qqaq=dirpe0xb9q8qiLsFr0=vr0=vr0dc8meaabaqaciaacaGaaeqabaqabeGadaaakeaacuWGHbqygaqeaaaa@2E0F@ (or b¯
 MathType@MTEF@5@5@+=feaafiart1ev1aaatCvAUfKttLearuWrP9MDH5MBPbIqV92AaeXatLxBI9gBaebbnrfifHhDYfgasaacH8akY=wiFfYdH8Gipec8Eeeu0xXdbba9frFj0=OqFfea0dXdd9vqai=hGuQ8kuc9pgc9s8qqaq=dirpe0xb9q8qiLsFr0=vr0=vr0dc8meaabaqaciaacaGaaeqabaqabeGadaaakeaacuWGIbGygaqeaaaa@2E11@ or c¯
 MathType@MTEF@5@5@+=feaafiart1ev1aaatCvAUfKttLearuWrP9MDH5MBPbIqV92AaeXatLxBI9gBaebbnrfifHhDYfgasaacH8akY=wiFfYdH8Gipec8Eeeu0xXdbba9frFj0=OqFfea0dXdd9vqai=hGuQ8kuc9pgc9s8qqaq=dirpe0xb9q8qiLsFr0=vr0=vr0dc8meaabaqaciaacaGaaeqabaqabeGadaaakeaacuWGJbWygaqeaaaa@2E13@ or d¯
 MathType@MTEF@5@5@+=feaafiart1ev1aaatCvAUfKttLearuWrP9MDH5MBPbIqV92AaeXatLxBI9gBaebbnrfifHhDYfgasaacH8akY=wiFfYdH8Gipec8Eeeu0xXdbba9frFj0=OqFfea0dXdd9vqai=hGuQ8kuc9pgc9s8qqaq=dirpe0xb9q8qiLsFr0=vr0=vr0dc8meaabaqaciaacaGaaeqabaqabeGadaaakeaacuWGKbazgaqeaaaa@2E15@) stands for *A *(*B *or *C *or *D*) = 0. We then calculate the numbers of exons and FTEs (denoted as ngj
 MathType@MTEF@5@5@+=feaafiart1ev1aaatCvAUfKttLearuWrP9MDH5MBPbIqV92AaeXatLxBI9gBaebbnrfifHhDYfgasaacH8akY=wiFfYdH8Gipec8Eeeu0xXdbba9frFj0=OqFfea0dXdd9vqai=hGuQ8kuc9pgc9s8qqaq=dirpe0xb9q8qiLsFr0=vr0=vr0dc8meaabaqaciaacaGaaeqabaqabeGadaaakeaacqWGUbGBdaWgaaWcbaGaem4zaC2aaSbaaWqaaiabdQgaQbqabaaaleqaaaaa@3129@ and ngjft
 MathType@MTEF@5@5@+=feaafiart1ev1aaatCvAUfKttLearuWrP9MDH5MBPbIqV92AaeXatLxBI9gBaebbnrfifHhDYfgasaacH8akY=wiFfYdH8Gipec8Eeeu0xXdbba9frFj0=OqFfea0dXdd9vqai=hGuQ8kuc9pgc9s8qqaq=dirpe0xb9q8qiLsFr0=vr0=vr0dc8meaabaqaciaacaGaaeqabaqabeGadaaakeaacqWGUbGBdaqhaaWcbaGaem4zaC2aaSbaaWqaaiabdQgaQbqabaaaleaacqWGMbGzcqWG0baDaaaaaa@33F0@) for each group *g*_*j*_. We then define a threshold *T *= ngjft
 MathType@MTEF@5@5@+=feaafiart1ev1aaatCvAUfKttLearuWrP9MDH5MBPbIqV92AaeXatLxBI9gBaebbnrfifHhDYfgasaacH8akY=wiFfYdH8Gipec8Eeeu0xXdbba9frFj0=OqFfea0dXdd9vqai=hGuQ8kuc9pgc9s8qqaq=dirpe0xb9q8qiLsFr0=vr0=vr0dc8meaabaqaciaacaGaaeqabaqabeGadaaakeaacqWGUbGBdaqhaaWcbaGaem4zaC2aaSbaaWqaaiabdQgaQbqabaaaleaacqWGMbGzcqWG0baDaaaaaa@33F0@/*EXP*(ngjft
 MathType@MTEF@5@5@+=feaafiart1ev1aaatCvAUfKttLearuWrP9MDH5MBPbIqV92AaeXatLxBI9gBaebbnrfifHhDYfgasaacH8akY=wiFfYdH8Gipec8Eeeu0xXdbba9frFj0=OqFfea0dXdd9vqai=hGuQ8kuc9pgc9s8qqaq=dirpe0xb9q8qiLsFr0=vr0=vr0dc8meaabaqaciaacaGaaeqabaqabeGadaaakeaacqWGUbGBdaqhaaWcbaGaem4zaC2aaSbaaWqaaiabdQgaQbqabaaaleaacqWGMbGzcqWG0baDaaaaaa@33F0@) and a Boolean function *f*_*ft*_(*g*_*j*_) as

fft(gj)={0,if T<21,if T≥2,j=0,1,...,15     (6)
 MathType@MTEF@5@5@+=feaafiart1ev1aaatCvAUfKttLearuWrP9MDH5MBPbIqV92AaeXatLxBI9gBaebbnrfifHhDYfgasaacH8akY=wiFfYdH8Gipec8Eeeu0xXdbba9frFj0=OqFfea0dXdd9vqai=hGuQ8kuc9pgc9s8qqaq=dirpe0xb9q8qiLsFr0=vr0=vr0dc8meaabaqaciaacaGaaeqabaqabeGadaaakeaacqWGMbGzdaWgaaWcbaGaemOzayMaemiDaqhabeaakiabcIcaOiabdEgaNnaaBaaaleaacqWGQbGAaeqaaOGaeiykaKIaeyypa0ZaaiqaaeaafaqaaeGacaaabaGaeGimaaJaeiilaWcabaGaeeyAaKMaeeOzayMaeeiiaaIaemivaqLaeyipaWJaeGOmaidabaGaeGymaeJaeiilaWcabaGaeeyAaKMaeeOzayMaeeiiaaIaemivaqLaeyyzImRaeGOmaidaaiabcYcaSiabdQgaQjabg2da9iabicdaWiabcYcaSiabigdaXiabcYcaSiabc6caUiabc6caUiabc6caUiabcYcaSiabigdaXiabiwda1aGaay5EaaGaaCzcaiaaxMaadaqadaqaaiabiAda2aGaayjkaiaawMcaaaaa@5978@

where *EXP*(ngjft
 MathType@MTEF@5@5@+=feaafiart1ev1aaatCvAUfKttLearuWrP9MDH5MBPbIqV92AaeXatLxBI9gBaebbnrfifHhDYfgasaacH8akY=wiFfYdH8Gipec8Eeeu0xXdbba9frFj0=OqFfea0dXdd9vqai=hGuQ8kuc9pgc9s8qqaq=dirpe0xb9q8qiLsFr0=vr0=vr0dc8meaabaqaciaacaGaaeqabaqabeGadaaakeaacqWGUbGBdaqhaaWcbaGaem4zaC2aaSbaaWqaaiabdQgaQbqabaaaleaacqWGMbGzcqWG0baDaaaaaa@33F0@) is the number of expected FTEs of *g*_*j *_and is computed as

Exp(ngjft)=ngj∑jngj×∑jngjft,j=0,1,...,15     (7)
 MathType@MTEF@5@5@+=feaafiart1ev1aaatCvAUfKttLearuWrP9MDH5MBPbIqV92AaeXatLxBI9gBaebbnrfifHhDYfgasaacH8akY=wiFfYdH8Gipec8Eeeu0xXdbba9frFj0=OqFfea0dXdd9vqai=hGuQ8kuc9pgc9s8qqaq=dirpe0xb9q8qiLsFr0=vr0=vr0dc8meaabaqaciaacaGaaeqabaqabeGadaaakeaacqWGfbqrcqWG4baEcqWGWbaCcqGGOaakcqWGUbGBdaqhaaWcbaGaem4zaC2aaSbaaWqaaiabdQgaQbqabaaaleaacqWGMbGzcqWG0baDaaGccqGGPaqkcqGH9aqpdaWcaaqaaiabd6gaUnaaBaaaleaacqWGNbWzdaWgaaadbaGaemOAaOgabeaaaSqabaaakeaadaaeqbqaaiabd6gaUnaaBaaaleaacqWGNbWzdaWgaaadbaGaemOAaOgabeaaaSqabaaabaGaemOAaOgabeqdcqGHris5aaaakiabgEna0oaaqafabaGaemOBa42aa0baaSqaaiabdEgaNnaaBaaameaacqWGQbGAaeqaaaWcbaGaemOzayMaemiDaqhaaaqaaiabdQgaQbqab0GaeyyeIuoakiabcYcaSiabdQgaQjabg2da9iabicdaWiabcYcaSiabigdaXiabcYcaSiabc6caUiabc6caUiabc6caUiabcYcaSiabigdaXiabiwda1iaaxMaacaWLjaWaaeWaaeaacqaI3aWnaiaawIcacaGLPaaaaaa@6432@

As stated in Equation (6), *f*_*ft*_(*g*_*j*_) = 1 indicates that *g*_*j *_includes 2 times more FTEs than expected. Using this Boolean function, we denote the Boolean values of fft(g0(a¯,b¯,c¯,d¯)), fft(g1(a¯,b¯,c¯,d)), fft(g2(a¯,b¯,c,d¯))
 MathType@MTEF@5@5@+=feaafiart1ev1aaatCvAUfKttLearuWrP9MDH5MBPbIqV92AaeXatLxBI9gBaebbnrfifHhDYfgasaacH8akY=wiFfYdH8Gipec8Eeeu0xXdbba9frFj0=OqFfea0dXdd9vqai=hGuQ8kuc9pgc9s8qqaq=dirpe0xb9q8qiLsFr0=vr0=vr0dc8meaabaqaciaacaGaaeqabaqabeGadaaakeaacqWGMbGzdaWgaaWcbaGaemOzayMaemiDaqhabeaakiabcIcaOiabdEgaNnaaBaaaleaacqaIWaamaeqaaOGaeiikaGIafmyyaeMbaebacqGGSaalcuWGIbGygaqeaiabcYcaSiqbdogaJzaaraGaeiilaWIafmizaqMbaebacqGGPaqkcqGGPaqkcqGGSaalcqqGGaaiieGacqWFMbGzdaWgaaWcbaGae8NzayMae8hDaqhabeaakiabcIcaOiabdEgaNnaaBaaaleaacqaIXaqmaeqaaOGaeiikaGIafmyyaeMbaebacqGGSaalcuWGIbGygaqeaiabcYcaSiqbdogaJzaaraGaeiilaWIaemizaqMaeiykaKIaeiykaKIaeiilaWIaeeiiaaIae8Nzay2aaSbaaSqaaiab=zgaMjab=rha0bqabaGccqGGOaakcqWGNbWzdaWgaaWcbaGaeGOmaidabeaakiabcIcaOiqbdggaHzaaraGaeiilaWIafmOyaiMbaebacqGGSaalcqWGJbWycqGGSaalcuWGKbazgaqeaiabcMcaPiabcMcaPaaa@66FB@, and fft(g3(a¯,b¯,c,d))
 MathType@MTEF@5@5@+=feaafiart1ev1aaatCvAUfKttLearuWrP9MDH5MBPbIqV92AaeXatLxBI9gBaebbnrfifHhDYfgasaacH8akY=wiFfYdH8Gipec8Eeeu0xXdbba9frFj0=OqFfea0dXdd9vqai=hGuQ8kuc9pgc9s8qqaq=dirpe0xb9q8qiLsFr0=vr0=vr0dc8meaabaqaciaacaGaaeqabaqabeGadaaakeaacqWGMbGzdaWgaaWcbaGaemOzayMaemiDaqhabeaakiabcIcaOiabdEgaNnaaBaaaleaacqaIZaWmaeqaaOGaeiikaGIafmyyaeMbaebacqGGSaalcuWGIbGygaqeaiabcYcaSiabdogaJjabcYcaSiabdsgaKjabcMcaPiabcMcaPaaa@3EEA@ as one. Therefore, *g*_0_~ *g*_3 _exons are FTE-rich. The Boolean algebraic expression can be represented as E=a¯b¯c¯d¯+a¯b¯c¯d+a¯b¯cd¯+a¯b¯cd
 MathType@MTEF@5@5@+=feaafiart1ev1aaatCvAUfKttLearuWrP9MDH5MBPbIqV92AaeXatLxBI9gBaebbnrfifHhDYfgasaacH8akY=wiFfYdH8Gipec8Eeeu0xXdbba9frFj0=OqFfea0dXdd9vqai=hGuQ8kuc9pgc9s8qqaq=dirpe0xb9q8qiLsFr0=vr0=vr0dc8meaabaqaciaacaGaaeqabaqabeGadaaakeaacqWGfbqrcqGH9aqpcuWGHbqygaqeaiqbdkgaIzaaraGafm4yamMbaebacuWGKbazgaqeaiabgUcaRiqbdggaHzaaraGafmOyaiMbaebacuWGJbWygaqeaiabdsgaKjabgUcaRiqbdggaHzaaraGafmOyaiMbaebacqWGJbWycuWGKbazgaqeaiabgUcaRiqbdggaHzaaraGafmOyaiMbaebacqWGJbWycqWGKbazaaa@476B@. We then use a 4-input (i.e., *A, B, C*, and *D*) Karnaugh map [23] to simplify *E *as  [see Additional file 1A]. The reduced expression reveals that a¯b¯
 MathType@MTEF@5@5@+=feaafiart1ev1aaatCvAUfKttLearuWrP9MDH5MBPbIqV92AaeXatLxBI9gBaebbnrfifHhDYfgasaacH8akY=wiFfYdH8Gipec8Eeeu0xXdbba9frFj0=OqFfea0dXdd9vqai=hGuQ8kuc9pgc9s8qqaq=dirpe0xb9q8qiLsFr0=vr0=vr0dc8meaabaqaciaacaGaaeqabaqabeGadaaakeaacuWGHbqygaqeaiqbdkgaIzaaraaaaa@2F74@ is the dominant condition (or FTE-rich condition) that result in an elevated FTE proportion under Equation (6), which is significantly larger than that of all applicable exons (15.1% vs. 5.1%, *P *< 0.01). Here the Karnaugh map is employed because it can analyze the interactions of up to six factors [23]. In contrast, the ANOVA analysis can hardly yield reliable results while analyzing interactions of more than three factors. If we use a more stringent threshold and set *T *as 2.5, then we can obtain a reduced Boolean expression *E' *as a¯b¯(c¯+d¯)
 MathType@MTEF@5@5@+=feaafiart1ev1aaatCvAUfKttLearuWrP9MDH5MBPbIqV92AaeXatLxBI9gBaebbnrfifHhDYfgasaacH8akY=wiFfYdH8Gipec8Eeeu0xXdbba9frFj0=OqFfea0dXdd9vqai=hGuQ8kuc9pgc9s8qqaq=dirpe0xb9q8qiLsFr0=vr0=vr0dc8meaabaqaciaacaGaaeqabaqabeGadaaakeaacuWGHbqygaqeaiqbdkgaIzaaraGaeiikaGIafm4yamMbaebacqGHRaWkcuWGKbazgaqeaiabcMcaPaaa@34D8@ [see additional file 1B]. The expression a¯b¯(c¯+d¯)
 MathType@MTEF@5@5@+=feaafiart1ev1aaatCvAUfKttLearuWrP9MDH5MBPbIqV92AaeXatLxBI9gBaebbnrfifHhDYfgasaacH8akY=wiFfYdH8Gipec8Eeeu0xXdbba9frFj0=OqFfea0dXdd9vqai=hGuQ8kuc9pgc9s8qqaq=dirpe0xb9q8qiLsFr0=vr0=vr0dc8meaabaqaciaacaGaaeqabaqabeGadaaakeaacuWGHbqygaqeaiqbdkgaIzaaraGaeiikaGIafm4yamMbaebacqGHRaWkcuWGKbazgaqeaiabcMcaPaaa@34D8@ means that two conditions are associated with elevated FTE proportions (i.e., a¯b¯c¯
 MathType@MTEF@5@5@+=feaafiart1ev1aaatCvAUfKttLearuWrP9MDH5MBPbIqV92AaeXatLxBI9gBaebbnrfifHhDYfgasaacH8akY=wiFfYdH8Gipec8Eeeu0xXdbba9frFj0=OqFfea0dXdd9vqai=hGuQ8kuc9pgc9s8qqaq=dirpe0xb9q8qiLsFr0=vr0=vr0dc8meaabaqaciaacaGaaeqabaqabeGadaaakeaacuWGHbqygaqeaiqbdkgaIzaaraGafm4yamMbaebaaaa@30DB@ or a¯b¯d¯
 MathType@MTEF@5@5@+=feaafiart1ev1aaatCvAUfKttLearuWrP9MDH5MBPbIqV92AaeXatLxBI9gBaebbnrfifHhDYfgasaacH8akY=wiFfYdH8Gipec8Eeeu0xXdbba9frFj0=OqFfea0dXdd9vqai=hGuQ8kuc9pgc9s8qqaq=dirpe0xb9q8qiLsFr0=vr0=vr0dc8meaabaqaciaacaGaaeqabaqabeGadaaakeaacuWGHbqygaqeaiqbdkgaIzaaraGafmizaqMbaebaaaa@30DD@), and both have to satisfy the condition that the Boolean values of *A *and *B *are both zero (i.e., a¯b¯
 MathType@MTEF@5@5@+=feaafiart1ev1aaatCvAUfKttLearuWrP9MDH5MBPbIqV92AaeXatLxBI9gBaebbnrfifHhDYfgasaacH8akY=wiFfYdH8Gipec8Eeeu0xXdbba9frFj0=OqFfea0dXdd9vqai=hGuQ8kuc9pgc9s8qqaq=dirpe0xb9q8qiLsFr0=vr0=vr0dc8meaabaqaciaacaGaaeqabaqabeGadaaakeaacuWGHbqygaqeaiqbdkgaIzaaraaaaa@2F74@). In other words, both *E *and *E' *indicate that *A *and *B *are the most dominant factors that affect the FTE proportions of ASEs. In addition, *E' *also indicates that either *C *or *D *has an additive effect to the A-B combination on FTE proportion. Moreover, if *T *is set as 3, we can obtain a reduced Boolean expression *E'' *= a¯b¯c¯
 MathType@MTEF@5@5@+=feaafiart1ev1aaatCvAUfKttLearuWrP9MDH5MBPbIqV92AaeXatLxBI9gBaebbnrfifHhDYfgasaacH8akY=wiFfYdH8Gipec8Eeeu0xXdbba9frFj0=OqFfea0dXdd9vqai=hGuQ8kuc9pgc9s8qqaq=dirpe0xb9q8qiLsFr0=vr0=vr0dc8meaabaqaciaacaGaaeqabaqabeGadaaakeaacuWGHbqygaqeaiqbdkgaIzaaraGafm4yamMbaebaaaa@30DB@ [see Additional file 1C]. The expression means that factor D is an insignificant variable when *T *= 3, and that the condition a¯b¯c¯
 MathType@MTEF@5@5@+=feaafiart1ev1aaatCvAUfKttLearuWrP9MDH5MBPbIqV92AaeXatLxBI9gBaebbnrfifHhDYfgasaacH8akY=wiFfYdH8Gipec8Eeeu0xXdbba9frFj0=OqFfea0dXdd9vqai=hGuQ8kuc9pgc9s8qqaq=dirpe0xb9q8qiLsFr0=vr0=vr0dc8meaabaqaciaacaGaaeqabaqabeGadaaakeaacuWGHbqygaqeaiqbdkgaIzaaraGafm4yamMbaebaaaa@30DB@ is more FTE-rich than a¯b¯d¯
 MathType@MTEF@5@5@+=feaafiart1ev1aaatCvAUfKttLearuWrP9MDH5MBPbIqV92AaeXatLxBI9gBaebbnrfifHhDYfgasaacH8akY=wiFfYdH8Gipec8Eeeu0xXdbba9frFj0=OqFfea0dXdd9vqai=hGuQ8kuc9pgc9s8qqaq=dirpe0xb9q8qiLsFr0=vr0=vr0dc8meaabaqaciaacaGaaeqabaqabeGadaaakeaacuWGHbqygaqeaiqbdkgaIzaaraGafmizaqMbaebaaaa@30DD@.

On the contrary, we can also explore which multiple factors are rich in exons that can pass the *K*_*A*_*/K*_*S *_ratio test. By performing the similar manner stated above, we can obtain a new Boolean expression *E*_*pass *_= *ab+bcd+acd *= *ab+cd*(*a+b*) [see Additional file 2]. The FTE proportion of *E*_*pass *_exons is only 0.7% (2 of 288 exons), which is much smaller than that of overall exons (*P *< 0.001). Furthermore, 100% of exons (199 of 199 exons) under condition *E*_*FTE *= 0 _= *abc+abd+bcd *(which is a sub-condition of *E*_*pass*_) pass the test. Notably, factors C and D are both negligible when exons are under condition *ab*. In other words, condition *ab*c¯d¯
 MathType@MTEF@5@5@+=feaafiart1ev1aaatCvAUfKttLearuWrP9MDH5MBPbIqV92AaeXatLxBI9gBaebbnrfifHhDYfgasaacH8akY=wiFfYdH8Gipec8Eeeu0xXdbba9frFj0=OqFfea0dXdd9vqai=hGuQ8kuc9pgc9s8qqaq=dirpe0xb9q8qiLsFr0=vr0=vr0dc8meaabaqaciaacaGaaeqabaqabeGadaaakeaacuWGJbWygaqeaiqbdsgaKzaaraaaaa@2F7C@ remains rich in passing-test exons even though such exons have low inclusion levels and low ESE motif frequencies. Therefore, the optimal condition for the *K*_*A*_*/K*_*S *_ratio test is *E*_*FTE *= 0_: *b (ac+ad+cd)*. In other words, an exon under condition *E*_*FTE *= 0 _may be assumed to be evolutionarily conserved. This may be useful when orthologous exons can not be found for the *K*_*A*_*/K*_*S *_ratio test.

**Figure 3:**
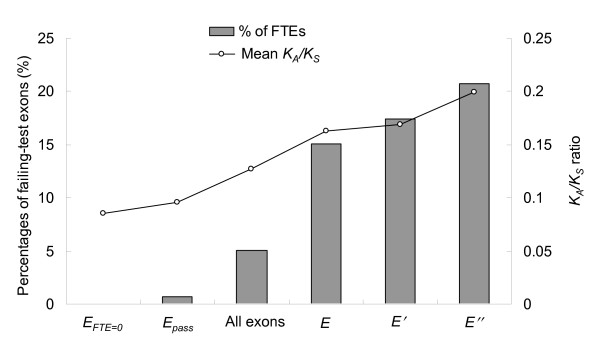
Summary of the FTE proportions and mean *K*_*A*_*/K*_*S *_ratios of ASEs under six different conditions: *E*_*FTE = 0 *_(*b(ac+ad+cd)*)*, E*_*pass *_(*ab+bcd+acd*), all applicable ASEs, *E *(a¯b¯
 MathType@MTEF@5@5@+=feaafiart1ev1aaatCvAUfKttLearuWrP9MDH5MBPbIqV92AaeXatLxBI9gBaebbnrfifHhDYfgasaacH8akY=wiFfYdH8Gipec8Eeeu0xXdbba9frFj0=OqFfea0dXdd9vqai=hGuQ8kuc9pgc9s8qqaq=dirpe0xb9q8qiLsFr0=vr0=vr0dc8meaabaqaciaacaGaaeqabaqabeGadaaakeaacuWGHbqygaqeaiqbdkgaIzaaraaaaa@2F74@),*E' *(a¯b¯(c¯+d¯)
 MathType@MTEF@5@5@+=feaafiart1ev1aaatCvAUfKttLearuWrP9MDH5MBPbIqV92AaeXatLxBI9gBaebbnrfifHhDYfgasaacH8akY=wiFfYdH8Gipec8Eeeu0xXdbba9frFj0=OqFfea0dXdd9vqai=hGuQ8kuc9pgc9s8qqaq=dirpe0xb9q8qiLsFr0=vr0=vr0dc8meaabaqaciaacaGaaeqabaqabeGadaaakeaacuWGHbqygaqeaiqbdkgaIzaaraGaeiikaGIafm4yamMbaebacqGHRaWkcuWGKbazgaqeaiabcMcaPaaa@34D8@) and *E" *(a¯b¯c¯
 MathType@MTEF@5@5@+=feaafiart1ev1aaatCvAUfKttLearuWrP9MDH5MBPbIqV92AaeXatLxBI9gBaebbnrfifHhDYfgasaacH8akY=wiFfYdH8Gipec8Eeeu0xXdbba9frFj0=OqFfea0dXdd9vqai=hGuQ8kuc9pgc9s8qqaq=dirpe0xb9q8qiLsFr0=vr0=vr0dc8meaabaqaciaacaGaaeqabaqabeGadaaakeaacuWGHbqygaqeaiqbdkgaIzaaraGafm4yamMbaebaaaa@30DB@). See text for more details.

Figure 3 summarizes the FTE proportions and mean *K*_*A*_*/K*_*S *_ratios of ASEs under five different conditions (*E*_*FTE *= 0_, *E*_*pass*_, *E*, *E'*, and *E''*). The FTE proportions of ASEs under these five conditions are all significantly different from that of all applicable exons. Furthermore, factors C and D have slight additive effects to factors A and B in affecting the FTE proportion, as can be observed in the insignificant differences in FTE proportions between conditions *E *and *E' *and between *E' *and *E" *(Figure 3).

In summary, our results indicate that length and protein domain overlapping are the two most important factors that affect evolutionary rates of ASEs. The two factors are correlated because longer exons have a higher probability of overlapping protein domains. Nevertheless, the additive effect of the two factors implies that length has some evolutionary effects that are irrelevant with protein domain (and vice versa). Intuitively, longer exons may include more regulatory signals than shorter ones, thus making them subject to stronger selective pressure. However, our results also show that ESEs have only minor effects on the evolutionary rates of ASEs. Although this may have resulted form inaccurate ESE predictions or limited ESE conservation, it appears likely that other regulatory signals (*e.g*. post-translational modification sites) and protein size/structure also play an important role in ASE evolution. Meanwhile, our results also imply that protein domain has evolutionary effects independent of exon length. This is understandable because very minor modifications in protein domains can result in dramatic structural/functional changes. Therefore, short, domain-overlapping exons that pass the *K*_*A*_*/K*_*S *_ratio test may be good targets for structural analysis for protein function inferences.

Meanwhile, our results also indicate that multiple exon features are interrelated in affecting evolutionary rates of ASEs. The observation implies that these exon features may be functionally correlated. Therefore, evolutionary and functional studies of ASEs should take into consideration the effects of multiple factors. In this sense, the method proposed in this study is a handy tool, for it not only distinguishes relative strength between factors, but also delineates combinational effects of multiple factors.

### Effects of exon features on ACEs

It is noteworthy that the ASEs analyzed so far are defined using human splicing patterns. Therefore, these ASEs in fact include both ACEs (73 out of 778 ASEs, ~ 10%) and lineage-specific ASEs (i.e., human-mouse orthologous exon pairs that are observed to be skipping in human but to be constitutive in mouse). To further explore the effects of exon features on ASE evolution, we retrieved ACEs from the ASD database [37] for the *K*_*A*_*/K*_*S *_ratio test. As shown in Table 3, ACEs have somewhat different evolutionary features from ASEs (Table 2). Similar to the results obtained from the ASE dataset, domain overlapping remains the most important, while ESE frequency insignificant in affecting the FTE proportion of ACEs. However, in the new dataset the influence of length is only marginally significant. Moreover, inclusion level also has a marginal effect on FTE proportion, which is different from the ASE-based results. These differences may have resulted from lineage-specific ASEs. Since these ASEs occurred after speciation events, they might have been under different selective pressure from that imposed on ACEs. Furthermore, we found that the ESE frequencies are positively related to inclusion level of ACEs, though the relationship is not significant [see Additional file 3].

It is surprising that the frequency of ESEs does not affect the FTE proportion in both datasets. ESEs are suggested to be enriched in CSEs [34], which have been shown to be under stronger selective pressure than ASEs [9,38,39]. It implies that exons with low *K*_*A*_*/K*_*S *_ratios have a higher frequency of ESEs. Therefore, ESE frequency is expected to be lower in FTEs than in passing-test exons. However, this scenario may be oversimplified for the following reasons. Firstly, other regulatory elements, including ESS (exonic splicing silencer), ISE (intronic splicing enhancer), and ISS (intronic splicing silencer), also participate in splicing regulation. Therefore, ESEs may not be the only (or the most important) factor that determines skipping of exons. Secondly, we have shown that inclusion level is only weakly related to FTE proportion. Even if ESEs can increase the inclusion level of their host exons, this ESE-inclusion level relationship may not ensure the association between ESE frequency and FTE proportion. Thirdly, ESEs are short and degenerate. An increased ESE frequency may not result in an elevated level of sequence conservation. Fourthly, the accuracy of predicted ESE motifs remains to be verified. Wang *et al *has reported that the motifs identified by ESEfinder do not significantly overlap with those detected by RESCUE-ESE [34]. Therefore, the relationship between ESE frequency and FTE proportion based on these predictions may be biased. Overall, ESE by itself appears to have no significant effects on the *K*_*A*_*/K*_*S *_ratios of ASEs. Nevertheless, the importance of regulatory elements in exon splicing has been well-established. It will be interesting to study the combinational effects of multiple regulatory elements (ESE, ESS, ISE, and ISS) on the *K*_*A*_*/K*_*S *_ratio test.

## Conclusion

In this study, we evaluate the relative strength and combinational effects of multiple exon features on evolutionary rates of ASEs. We have reached the following conclusions: Firstly, non-applicable exons will bias the exon feature-evolutionary rate relationship and should be excluded from analysis. Secondly, length and protein domain overlapping individually, and also additively, have the greatest effects on ASE evolutionary rates. However, ACEs shown a somewhat different trend in that inclusion level and length both have a marginally significant effect on FTE proportion. The difference possibly has resulted from differences between lineage-specific ASEs and ACEs. Thirdly, ESE motif frequency is likely to be the weakest of the four factors studied. This is surprising because ESEs are regarded important for alternative splicing regulation and evolutionarily conserved. Fourthly, length and protein domain overlapping have additive effects on evolutionary rates. Finally, we have identified a combination of ASE features (*E*_*FTE *= 0_) that characterize evolutionarily conserved exons. This can be useful when no orthologous exons are available for comparative analysis.

## Methods

### Extraction of human-mouse orthologous exon pairs

For the ASE dataset, well-annotated human CSEs and ASEs were downloaded from the online database ASAP (the Alternative Splicing Annotation Project [40,41]). By mapping the ASAP-provided homolog table to the ASAP genomic data set or the corresponding mouse UniGene EST sequences (March 2005 [42]), human CSEs and their orthologous mouse exonic sequences were retrieved. Since the mouse orthologues of human ASEs were not available from ASAP, we Blastn-aligned the human ASEs plus two flanking exons against the mouse UniGene database and the mouse genomic sequences. Mouse exons that had a = 70% sequence identity to the full lengths of human exon queries were extracted. A total of 778 human ASEs, including 508 major-form exons, 20 minor-form exons, and 250 undetermined-form exons, were paired with their mouse orthologs. The classification of major-form (included in at least two thirds of the EST counts), minor-form (skipped in at least two thirds of the EST counts), and undetermined-form (in the intermediate case, or ≤ 5 ESTs in total) exons was provided by ASAP (also defined in Modrek and Lees' study (2003)). The minor-form and undetermined-form exons were then merged to form the non-major-form exon group.

For the ACE dataset, ACEs were downloaded from the online database ASD (Alternative Splicing Database, AltSplice Human Release 2 based on Ensembl 27.35a.1 and AltSplice Mouse Release 2 based on Ensembl 27.33c.1 [37,43]). We used EST (i.e., the human UniGene EST database) counts to classify human major-form and non-major-form ACEs by the definition stated above. The sequences of exons analyzed in this study (including CSEs, ASEs, and ACEs) are available [44].

### The *K*_*A*_*/K*_*S *_ratio test

For the *K*_*A*_*/K*_*S *_ratio analysis of orthologous exon pairs, we performed the following procedures: (i) calculating the numbers of synonymous and non-synonymous sites, *K*_*A*_, *K*_*S*_, and *K*_*A*_*/K*_*S *_values, using the yn00 program of the PAML package [45,46]. The exon pairs were aligned in FASTA format and checked for correct reading frame before submitting to the PAML program; (ii) creating two-way contingency tables with rows comprising numbers of synonymous and non-synonymous sites and columns comprising numbers of changed and unchanged sites; and (iii) testing the independence between the numbers of changed synonymous and non-synonymous sites using one-tailed Fisher's exact test. The Fisher's exact test was performed in *R *statistics system [47]. If the ratio *K*_*A*_*/K*_*S *_is significantly smaller than one at 5% level (*P *< 0.05), it stands for that the orthologous exon pairs tested are under strong negative selection. Such exons are termed "passing-test exons"; otherwise the exons are termed FTEs (failing-test exons). The FTEs are likely under positive selection or relaxed negative selection, which may accelerate changes at the amino acid level [12,13].

### Identification of ESE motifs

Three programs were used for ESE motif prediction: ESEfinder, RESCUE-ESE and PESE, all with default parameters. The three programs yielded somewhat different results. ESEfinder uses weight matrices to predict ESE motifs that are responsive to four human SR proteins (SF2/ASF, SC35, SRp40 and SRp55). The matrices are based on frequency values derived from alignments of SELEX-derived sequences [28]. In contrast, both RESCUE-ESE [29-31] and PESE [32,33] are *ab initio *methods. The two *ab initio *methods differ in that the former identifies hexamers preferentially associated with CSEs with weak splice sites, while the latter detects octamers overrepresented in non-coding exons compared with the 5'UTR of intronless genes and pseudo exons [34].

### Determination of low/high ESE motif frequencies of exons

Using ESEfinder [28] with default parameters, we identified ESE motifs in the studied ASEs. For each exon e_*i*_, we then calculated the total number of these four identified ESE motifs neiESE
 MathType@MTEF@5@5@+=feaafiart1ev1aaatCvAUfKttLearuWrP9MDH5MBPbIqV92AaeXatLxBI9gBaebbnrfifHhDYfgasaacH8akY=wiFfYdH8Gipec8Eeeu0xXdbba9frFj0=OqFfea0dXdd9vqai=hGuQ8kuc9pgc9s8qqaq=dirpe0xb9q8qiLsFr0=vr0=vr0dc8meaabaqaciaacaGaaeqabaqabeGadaaakeaacqWGUbGBdaqhaaWcbaGaemyzau2aaSbaaWqaaiabdMgaPbqabaaaleaacqWGfbqrcqWGtbWucqWGfbqraaaaaa@3479@. Suppose that the length of e_*i *_is ℓei
 MathType@MTEF@5@5@+=feaafiart1ev1aaatCvAUfKttLearuWrP9MDH5MBPbIqV92AaeXatLxBI9gBaebbnrfifHhDYfgasaacH8akY=wiFfYdH8Gipec8Eeeu0xXdbba9frFj0=OqFfea0dXdd9vqai=hGuQ8kuc9pgc9s8qqaq=dirpe0xb9q8qiLsFr0=vr0=vr0dc8meaabaqaciaacaGaaeqabaqabeGadaaakeaacqWItecBdaWgaaWcbaacbaGae8xzau2aaSbaaWqaaiab=LgaPbqabaaaleqaaaaa@30F0@ the expected ESE motif number of e_*i*_, *EXP*(neiESE
 MathType@MTEF@5@5@+=feaafiart1ev1aaatCvAUfKttLearuWrP9MDH5MBPbIqV92AaeXatLxBI9gBaebbnrfifHhDYfgasaacH8akY=wiFfYdH8Gipec8Eeeu0xXdbba9frFj0=OqFfea0dXdd9vqai=hGuQ8kuc9pgc9s8qqaq=dirpe0xb9q8qiLsFr0=vr0=vr0dc8meaabaqaciaacaGaaeqabaqabeGadaaakeaacqWGUbGBdaqhaaWcbaGaeeyzau2aaSbaaWqaaiabbMgaPbqabaaaleaacqWGfbqrcqWGtbWucqWGfbqraaaaaa@3475@) can be computed as

EXP(neiESE)=ℓei∑iℓei×∑ineiESE
 MathType@MTEF@5@5@+=feaafiart1ev1aaatCvAUfKttLearuWrP9MDH5MBPbIqV92AaeXatLxBI9gBaebbnrfifHhDYfgasaacH8akY=wiFfYdH8Gipec8Eeeu0xXdbba9frFj0=OqFfea0dXdd9vqai=hGuQ8kuc9pgc9s8qqaq=dirpe0xb9q8qiLsFr0=vr0=vr0dc8meaabaqaciaacaGaaeqabaqabeGadaaakeaacqWGfbqrcqWGybawcqWGqbaucqGGOaakcqqGUbGBdaqhaaWcbaGaeeyzau2aaSbaaWqaaiabbMgaPbqabaaaleaacqWGfbqrcqWGtbWucqWGfbqraaGccqGGPaqkcqGH9aqpdaWcaaqaaiabloriSnaaBaaaleaacqqGLbqzdaWgaaadbaGaeeyAaKgabeaaaSqabaaakeaadaaeqbqaaiabloriSnaaBaaaleaacqqGLbqzdaWgaaadbaGaeeyAaKgabeaaaSqabaaabaGaemyAaKgabeqdcqGHris5aaaakiabgEna0oaaqafabaGaemOBa42aa0baaSqaaiabbwgaLnaaBaaameaacqqGPbqAaeqaaaWcbaGaemyrauKaem4uamLaemyraueaaaqaaiabdMgaPbqab0GaeyyeIuoaaaa@5412@

If neiESE
 MathType@MTEF@5@5@+=feaafiart1ev1aaatCvAUfKttLearuWrP9MDH5MBPbIqV92AaeXatLxBI9gBaebbnrfifHhDYfgasaacH8akY=wiFfYdH8Gipec8Eeeu0xXdbba9frFj0=OqFfea0dXdd9vqai=hGuQ8kuc9pgc9s8qqaq=dirpe0xb9q8qiLsFr0=vr0=vr0dc8meaabaqaciaacaGaaeqabaqabeGadaaakeaacqWGUbGBdaqhaaWcbaGaemyzau2aaSbaaWqaaiabdMgaPbqabaaaleaacqWGfbqrcqWGtbWucqWGfbqraaaaaa@3479@ > *EXP*(neiESE
 MathType@MTEF@5@5@+=feaafiart1ev1aaatCvAUfKttLearuWrP9MDH5MBPbIqV92AaeXatLxBI9gBaebbnrfifHhDYfgasaacH8akY=wiFfYdH8Gipec8Eeeu0xXdbba9frFj0=OqFfea0dXdd9vqai=hGuQ8kuc9pgc9s8qqaq=dirpe0xb9q8qiLsFr0=vr0=vr0dc8meaabaqaciaacaGaaeqabaqabeGadaaakeaacqWGUbGBdaqhaaWcbaGaeeyzau2aaSbaaWqaaiabbMgaPbqabaaaleaacqWGfbqrcqWGtbWucqWGfbqraaaaaa@3475@)then we define that e_*i *_has high ESE motif frequency; otherwise e_*i *_has low ESE motif frequency.

## Abbreviations

AS – Alternative Splicing

ASEs – Alternatively Spliced Exons

CSEs – Constitutively Spliced Exons

ACEs – Alternative Conserved Exons

The *K*_*A*_*/K*_*S *_ratio test – the non-synonymous to synonymous substation rate ratio test

FTEs – Failing-Test Exons

ESE – Exonic Splicing Enhancer;

## Authors' contributions

TJC designed the method and FCC analyzed the data. Both authors read and approved the final manuscript.

## Supplementary Material

Additional File 1 The reduced results of the Karnaugh map for four Boolean expressions of ASE features. (A) Condition *E *(= a¯b¯
 MathType@MTEF@5@5@+=feaafiart1ev1aaatCvAUfKttLearuWrP9MDH5MBPbIqV92AaeXatLxBI9gBaebbnrfifHhDYfgasaacH8akY=wiFfYdH8Gipec8Eeeu0xXdbba9frFj0=OqFfea0dXdd9vqai=hGuQ8kuc9pgc9s8qqaq=dirpe0xb9q8qiLsFr0=vr0=vr0dc8meaabaqaciaacaGaaeqabaqabeGadaaakeaacuWGHbqygaqeaiqbdkgaIzaaraaaaa@2F74@); (B) *E' *(= a¯b¯c¯
 MathType@MTEF@5@5@+=feaafiart1ev1aaatCvAUfKttLearuWrP9MDH5MBPbIqV92AaeXatLxBI9gBaebbnrfifHhDYfgasaacH8akY=wiFfYdH8Gipec8Eeeu0xXdbba9frFj0=OqFfea0dXdd9vqai=hGuQ8kuc9pgc9s8qqaq=dirpe0xb9q8qiLsFr0=vr0=vr0dc8meaabaqaciaacaGaaeqabaqabeGadaaakeaacuWGHbqygaqeaiqbdkgaIzaaraGafm4yamMbaebaaaa@30DB@ + a¯b¯d¯
 MathType@MTEF@5@5@+=feaafiart1ev1aaatCvAUfKttLearuWrP9MDH5MBPbIqV92AaeXatLxBI9gBaebbnrfifHhDYfgasaacH8akY=wiFfYdH8Gipec8Eeeu0xXdbba9frFj0=OqFfea0dXdd9vqai=hGuQ8kuc9pgc9s8qqaq=dirpe0xb9q8qiLsFr0=vr0=vr0dc8meaabaqaciaacaGaaeqabaqabeGadaaakeaacuWGHbqygaqeaiqbdkgaIzaaraGafmizaqMbaebaaaa@30DD@); (C) *E'' *= a¯b¯c¯
 MathType@MTEF@5@5@+=feaafiart1ev1aaatCvAUfKttLearuWrP9MDH5MBPbIqV92AaeXatLxBI9gBaebbnrfifHhDYfgasaacH8akY=wiFfYdH8Gipec8Eeeu0xXdbba9frFj0=OqFfea0dXdd9vqai=hGuQ8kuc9pgc9s8qqaq=dirpe0xb9q8qiLsFr0=vr0=vr0dc8meaabaqaciaacaGaaeqabaqabeGadaaakeaacuWGHbqygaqeaiqbdkgaIzaaraGafm4yamMbaebaaaa@30DB@ Click here for file

Additional File 2 Procedure of exploring which multiple factors are rich in exons that can pass the *K*_*A*_*/K*_*S *_ratio test. Click here for file

Additional File 3 Properties and evolutionary features (*K*_*A*_, *K*_*S*_, and *K*_*A*_*/K*_*S *_values) of the retrieved human-mouse orthologous exons: CCEs, major-form ACEs, and non-major-form ACEs. Here, CCEs (ACEs) are the human-mouse orthologous exon pairs that are observed to be constitutive (skipping) in both human and mouse. CCEs and ACEs are both retrieved from the ASD database. Click here for file

## Figures and Tables

**Table 1:** The retrieved human constitutively spliced exons (CSEs) and alternatively spiced exons (ASEs).

	**CSEs**	**ASEs**	
		
		Major	Non-major
No. of exons analyzed	4,630	508	270
No. of exons with length ≤ 75 bp	807 (17.4%)	97 (19.1%)	71 (26.3%)
No. of exons with divergence ≤ 5%	264 (5.7%)	59 (11.6%)	47 (17.4%)
*No. of exons with length ≤ 75 bp or divergence ≤ 5%	974 (21.0%)	135 (26.6%)	92 (34.1%)

* Defined as "non-applicable exons".

**Table 2:** The effects of single exon features on the results of the *K*_*A*_*/K*_*S *_ratio test on applicable ASEs.

Exon features		Mean *K*_*A*_*/K*_*S*_	# Pass	# Fail	% Fail	*P*-value*
**A: length**	≤ 100 bp	0.131	127	16	11.2	< 0.01
	> 100 bp	0.126	396	12	2.9	

**B: domain-overlapping**	No	0.166	259	25	8.8	< 0.01
	Yes	0.087	264	3	1.1	

**C: inclusion level**	Non-major	0.148	165	13	7.3	> 0.1
	Major	0.118	358	15	4.0	

**D: ESE frequency**	Low	0.134	248	17	6.4	> 0.1
	High	0.121	275	11	3.8	

A: > 100 bp and B: No		0.167	180	11	5.8	> 0.1
A: ≤ 100 bp and B: Yes		0.072	48	2	4.0	

*Comparison of numbers of passing-test and failing-test exons by two-tailed Fisher's exact test.

**Table 3:** The effects of four exon features on the results of the *K*_*A*_*/K*_*S *_ratio test on applicable ACEs.

Exon features		Mean *K*_*A*_*/K*_*S*_	# Pass	# Fail	% Fail	*P*-value*
**A: length**	≤ 100 bp	0.300	65	14	17.7	< 0.05
	> 100 bp	0.172	165	15	8.3	

**B: domain-overlapping**	No	0.293	64	16	20.0	< 0.01
	Yes	0.174	166	13	7.26	

**C: inclusion level**	Non-major	0.267	58	13	18.3	< 0.05
	Major	0.190	172	16	8.5	

**D: ESE frequency**	Low	0.226	116	16	12.1	> 0.1
by ESEfinder	High	0.196	114	13	10.2	
by RESCUE-ESE	Low	0.186	128	13	9.2	> 0.1
	High	0.241	102	16	13.6	
by PESE	Low	0.178	127	12	8.6	> 0.1
	High	0.249	103	17	14.2	

*Comparison of numbers of passing-test and failing-test exons by two-tailed Fisher's exact test.
