# Supplementary material for: The effects of multiple features of alternatively spliced exons on the KA/KS ratio test
Source: BMC Bioinformatics. 2006 May 19;7:259. doi: 10.1186/1471-2105-7-259 (PMC1526763; doi:10.1186/1471-2105-7-259)
Supplement: Additional File 1 — The reduced results of the Karnaugh map for four Boolean expressions of ASE features. (A) Condition E (= a¯b¯ MathType@MTEF@5@5@+=feaafiart1ev1aaatCvAUfKttLearuWrP9MDH5MBPbIqV92AaeXatLxBI9gBaebbnrfifHhDYfgasaacH8akY=wiFfYdH8Gipec8Eeeu0xXdbba9frFj0=OqFfea0dXdd9vqai=hGuQ8kuc9pgc9s8qqaq=dirpe0xb9q8qiLsFr0=vr0=vr0dc8meaabaqaciaacaGaaeqabaqabeGadaaakeaacuWGHbqygaqeaiqbdkgaIzaaraaaaa@2F74@); (B) E' (= a¯b¯c¯ MathType@MTEF@5@5@+=feaafiart1ev1aaatCvAUfKttLearuWrP9MDH5MBPbIqV92AaeXatLxBI9gBaebbnrfifHhDYfgasaacH8akY=wiFfYdH8Gipec8Eeeu0xXdbba9frFj0=OqFfea0dXdd9vqai=hGuQ8kuc9pgc9s8qqaq=dirpe0xb9q8qiLsFr0=vr0=vr0dc8meaabaqaciaacaGaaeqabaqabeGadaaakeaacuWGHbqygaqeaiqbdkgaIzaaraGafm4yamMbaebaaaa@30DB@ + a¯b¯d¯ MathType@MTEF@5@5@+=feaafiart1ev1aaatCvAUfKttLearuWrP9MDH5MBPbIqV92AaeXatLxBI9gBaebbnrfifHhDYfgasaacH8akY=wiFfYdH8Gipec8Eeeu0xXdbba9frFj0=OqFfea0dXdd9vqai=hGuQ8kuc9pgc9s8qqaq=dirpe0xb9q8qiLsFr0=vr0=vr0dc8meaabaqaciaacaGaaeqabaqabeGadaaakeaacuWGHbqygaqeaiqbdkgaIzaaraGafmizaqMbaebaaaa@30DD@); (C) E'' = a¯b¯c¯ MathType@MTEF@5@5@+=feaafiart1ev1aaatCvAUfKttLearuWrP9MDH5MBPbIqV92AaeXatLxBI9gBaebbnrfifHhDYfgasaacH8akY=wiFfYdH8Gipec8Eeeu0xXdbba9frFj0=OqFfea0dXdd9vqai=hGuQ8kuc9pgc9s8qqaq=dirpe0xb9q8qiLsFr0=vr0=vr0dc8meaabaqaciaacaGaaeqabaqabeGadaaakeaacuWGHbqygaqeaiqbdkgaIzaaraGafm4yamMbaebaaaa@30DB@ [file 1471-2105-7-259-S1.doc]

Supplementary Table 1. Properties and evolutionary features (*K_A_*, *K_S_*, and *K_A_/K_S_* values) of the retrieved human-mouse orthologous exons: CCEs^*^, major-form ACEs^*^, and non-major-form ACEs^*^.

|  | | Inclusion level | | |
| --- | --- | --- | --- | --- |
|  | | CCEs | ACEs | |
|  | |  | Major | Non-major |
| Number of exons analyzed | | 21701 | 262 | 116 |
| Average length (bp) | | 132 | 119 | 118 |
| ESE frequency | ESEfinder | 0.116 | 0.111 | 0.109 |
|  | RESCUE-ESE | 0.105 | 0.097 | 0.093 |
|  | PESE | 0.189 | 0.175 | 0.171 |
| Median *K_A_* value | | 0.028 | 0.034 | 0.034 |
| Median *K_S_* value | | 0.599 | 0.427 | 0.282 |
| Median *K_A_/K_S_* ratio | | 0.043 | 0.080 | 0.132 |
| Fail-testing exon (FTE) rate (%) | | 6.06 | 21.37 | 36.21 |

^*^CCEs (ACEs) are the human-mouse orthologous exon pairs that are observed to be constitutive (skipping) in both human and mouse. CCEs and ACEs are both retrieved from the ASD database.
