# Supplementary material for: The effects of multiple features of alternatively spliced exons on the KA/KS ratio test
Source: BMC Bioinformatics. 2006 May 19;7:259. doi: 10.1186/1471-2105-7-259 (PMC1526763; doi:10.1186/1471-2105-7-259)
Supplement: Additional File 2 — Procedure of exploring which multiple factors are rich in exons that can pass the KA/KS ratio test. [file 1471-2105-7-259-S2.doc]

(A) =$\left\{\begin{matrix} 0, & \text{if }T<2 \\ 1, & \text{if }T\geq2 \end{matrix}\right.$, *j* = 0,1,…,15

| AB  CD | 00 | 01 | 11 | 10 |
| --- | --- | --- | --- | --- |
| 00 | 1 | 0 | 0 | 0 |
| 01 | 1 | 0 | 0 | 0 |
| 11 | 1 | 0 | 0 | 0 |
| 10 | 1 | 0 | 0 | 0 |

(B) =$\left\{\begin{matrix} 0, & \text{if }T<2.5 \\ 1, & \text{if }T\geq2.5 \end{matrix}\right.$, *j* = 0,1,…,15

| AB  CD | 00 | 01 | 11 | 10 |
| --- | --- | --- | --- | --- |
| 00 | 1 | 0 | 0 | 0 |
| 01 | 1 | 0 | 0 | 0 |
| 11 | 0 | 0 | 0 | 0 |
| 10 | 1 | 0 | 0 | 0 |

(C) =$\left\{\begin{matrix} 0, & \text{if }T<3 \\ 1, & \text{if }T\geq3 \end{matrix}\right.$, *j* = 0,1,…,15

| AB  CD | 00 | 01 | 11 | 10 |
| --- | --- | --- | --- | --- |
| 00 | 1 | 0 | 0 | 0 |
| 01 | 1 | 0 | 0 | 0 |
| 11 | 0 | 0 | 0 | 0 |
| 10 | 0 | 0 | 0 | 0 |

Supplementary Figure 1. The reduced results of the Karnaugh map for four Boolean expressions of ASE features. (A) Condition *E* (= $\overline{a}\overline{b}$); (B) *E’* (= +$\overline{a}\overline{b}\overline{d}$); (C) *E’’* (= ).
