# Supplementary material for: The effects of multiple features of alternatively spliced exons on the KA/KS ratio test
Source: BMC Bioinformatics. 2006 May 19;7:259. doi: 10.1186/1471-2105-7-259 (PMC1526763; doi:10.1186/1471-2105-7-259)
Supplement: Additional File 3 — Properties and evolutionary features (KA, KS, and KA/KS values) of the retrieved human-mouse orthologous exons: CCEs, major-form ACEs, and non-major-form ACEs. Here, CCEs (ACEs) are the human-mouse orthologous exon pairs that are observed to be constitutive (skipping) in both human and mouse. CCEs and ACEs are both retrieved from the ASD database. [file 1471-2105-7-259-S3.doc]

**Supplementary Note**

Which multiple factors are rich in exons that can pass the *K_A_/K_S_* ratio test?

If is redefined as

$f_{ft}(g_{j})$=$\left\{\begin{matrix} 0, & \text{if }T\geq\text{ 1 or }n_{g_{j}}<10 \\ 1, & \text{if }T<1\text{ } \end{matrix}\right.$, *j* = 0,1,…,15 (1)

then we can explore which multiple factors are rich in exons that can pass the *K_A_/K_S_* ratio test. According to Equation (1) and the corresponding **Karnaugh map (see Fig. 1)**, we can obtain a new Boolean expression *E_pass_* = *ab+bcd+acd* = *ab+cd*(*a+b*).

| AB  CD | 00 | 01 | 11 | 10 |
| --- | --- | --- | --- | --- |
| 00 | 0 | 0 | 1 | 0 |
| 01 | 0 | 0 | 1 | 0 |
| 11 | 0 | 1 | 1 | 1 |
| 10 | 0 | 0 | 1 | 0 |

Figure 1. **Karnaugh map of** *E_pass_* (= *ab+bcd+acd*).
